# Editorial: An integrated positive psychology approach into counseling in different settings

**DOI:** 10.3389/fpsyg.2023.1205309

**Published:** 2023-05-10

**Authors:** Christos Pezirkianidis, Anastassios Stalikas, Panagiotis Parpottas

**Affiliations:** ^1^Lab of Positive Psychology, Department of Psychology, Panteion University of Social and Political Sciences, Athens, Greece; ^2^Department of Social and Behavioral Sciences, European University of Cyprus, Engomi, Cyprus

**Keywords:** positive psychology, counseling, integration, wellbeing, counseling psychology

## Introduction

Positive psychology (PP) is the scientific field that focuses on revealing, understanding, and reinforcing the factors that make individuals and systems flourish. At first, PP research gave emphasis on individual's positive experiences and characteristics (Pezirkianidis and Stalikas, 2020). Later on, the second research wave focused on the dialectic relationship between the positive and negative aspects of one's life, proposing that wellbeing can only be achieved through overcoming suffering (Wong, 2019). Recently, the third wave of research suggested PP to become more interdisciplinary and multicultural and incorporate systems' principles (Lomas et al., 2021).

Counseling psychology (CP) and PP share their roots in humanistic psychology. Also, CP focuses on a positive orientation toward individual development, mental health promotion and prevention rather than pathology (Malikiosi-Loizos and Ivey, 2012). However, while US counseling psychologists have incorporated PP principles, European ones hesitate to engage with strength-based approaches and use PP theories and techniques (Steffen et al., 2015).

At the same time, more and more approaches in CP have emerged focusing on building positive qualities and making use of the empirically tested positive psychology interventions (PPIs; Carr et al., 2021). These models focus on the promotion of clients' wellbeing and, simultaneously, on symptom alleviation (Jankowski et al., 2020). Counseling practitioners can offer their skills and knowledge as a fertile ground for the application of such models, while at the same time they can benefit by integrating new techniques in their counseling practice (D'raven and Pasha-Zaidi, 2014).

## Overview of studies

Eleven articles in this Research Topic capture different aspects of integrating the PP approach into counseling for children, youth, university students and adults. These articles provide a systematic review of the current literature (Galanakis and Tsitouri; Kritikou and Giovazolias; Pezirkianidis et al.), study the relationships between PP variables and flourishing factors that connect to counseling needs (Argyrides and Anastasiades; Liu et al.; Min et al.; Tuxunjiang et al.), and evaluate interventions (Karampas et al.; Kounenou et al.; Touloupis and Athanasiades; Zhang et al.) to highlight the added value of PP into counseling practice.

### A positive psychology approach into counseling for children and youth

PP has been extensively studied in the context of early intervention, positive prevention and mental health promotion in children and adolescents, proving to have an added value on counseling with typically developing children and young clients facing psychological problems or being at-risk (Owens and Waters, 2020).

Two contributions in this Research Topic focus on the integration of PP principles into counseling with children and youth. Touloupis and Athanasiades' paper which examined the effectiveness of a cyberbullying prevention program focusing on enhancing self-esteem among elementary school students, found that students' cyberbullying engagement decreased after the intervention mainly because of changes in self-esteem levels. Zhang et al. applied a group counseling intervention to orphans and vulnerable children focusing on enhancing positive interpersonal relationship skills and found increased social support and posttraumatic growth after the intervention. Both studies highlight the importance of PP principles on increasing the effectiveness of counseling interventions to children and youth.

### The integration of PP into university student counseling

In the previous two decades, many studies have focused on the effects of PP variables on wellbeing indices of university students (Carr et al., 2021). Four articles in the present Research Topic address this issue. Kritikou and Giovazolias systematically reviewed the literature and found that several PP variables (i.e., self-efficacy, positive learning climate, and positive interpersonal relationships) affect emotion regulation, academic buoyancy, and academic adjustment of university students. Min et al. found that post-graduate students' self-compassion predicts positive and negative wellbeing indices, while non-professional help-seeking behavior partially mediates this relationship. Kounenou et al. implemented a multicomponent PPI and demonstrated its effects on increased experience of positive emotions among undergraduate students. Finally, Karampas et al. examined the effectiveness of an innovative counseling intervention aiming at changing university students' stress mindset. Their findings indicate an increase in “stress-is-enhancing” mindset, life satisfaction, and self-efficacy against stress, as well as a decrease in “stress-is-debilitating” mindset. Taking everything into account, PP variables explain several positive outcomes concerning university students' academic and psychological wellbeing and seem promising in enriching counseling practice with this population.

### Counseling services for adults through the lens of PP

Research has also emphasized on the effects of PP variables on many aspects of adult life cycle. Five articles in this Research Topic address adult counseling issues. Galanakis and Tsitouri's systematic literature review offered evidence that the job demands-resources model predicts employee wellbeing. Similarly, Liu et al. provided evidence on the effects of PP variables, namely psychological capital, on work outcomes (humanistic care ability of nurses). Moreover, a literature review by Pezirkianidis et al., found that adult friendship predicts wellbeing components based on the PERMA model and recommended the implementation of positive friendship interventions. Tuxunjiang et al. investigated protective psychological factors of pregnant women and found that self-efficacy, perceived social support and resilience act protectively against anxiety and stress. Finally, Argyrides and Anastasiades focused on positive eating habits and found influences of gender and body mass on intuitive eating behaviors among adults.

## Conclusions

PP can contribute to CP in many ways and in different contexts, e.g., school, work, university, and everyday life. The gradual integration of its principles and interventions could enhance the effectiveness of the counseling process and, in this way, CP could recommit toward human flourishing and actualization. However, the need to continue building PP scholarship and educating counseling psychologists on how to integrate PP principles and techniques still stands.

## Author contributions

CP wrote the article. AS and PP supervised all stages of writing and provided feedback. All authors contributed to the article and approved the submitted version.
